# Klebsiella aerogenes Discitis in a 78-Year-Old Male Patient With End-Stage Renal Disease on Hemodialysis: A Case Report

**DOI:** 10.7759/cureus.109802

**Published:** 2026-05-28

**Authors:** Khaled Abu Baker, SriYoshitha Inala, SyedUmairBin Uzair, Murad Haddad, Sanil Thomas

**Affiliations:** 1 Internal Medicine, HCA Florida North Florida Hospital, Gainesville, USA; 2 Infectious Diseases, HCA Florida North Florida Hospital, Gainesville, USA

**Keywords:** anti-microbial agents, end-stage renal disease (esrd), hemodialysis access, klebsiella aerogenes infection, vertebral discitis

## Abstract

Vertebral osteomyelitis is a well-recognized complication in hemodialysis patients, though it is most commonly attributed to Gram-positive organisms, such as *Staphylococcus aureus*. Gram-negative pathogens, while less frequently implicated, are increasingly appreciated in this population given the unique confluence of immune dysfunction, recurrent vascular access manipulation, and frequent healthcare exposure. We describe the case of a 78-year-old man on hemodialysis who presented with progressive lower back pain and was ultimately found to have L4-L5 discitis caused by *Klebsiella aerogenes*. The diagnosis required repeat disc aspiration after initial cultures were unrevealing. The source of infection was not definitively established; while hematogenous seeding from the hemodialysis vascular access was considered given the patient's recurrent bloodstream exposure, a genitourinary origin could not be excluded, as *Klebsiella *species are well-known colonizers of the urinary tract and a recognized source of Gram-negative bacteremia. He responded well to a prolonged course of targeted intravenous antibiotic therapy. This case underscores the importance of maintaining a broad differential for vertebral osteomyelitis in hemodialysis patients, considering both vascular access-related and genitourinary sources of infection, and pursuing repeat sampling when clinical suspicion persists despite a negative initial workup.

## Introduction

Vertebral osteomyelitis in hemodialysis patients carries significant morbidity and mortality. While *Staphylococcus aureus* is the predominant pathogen, Gram-negative organisms are increasingly recognized. *Klebsiella aerogenes* is a rare cause of discitis with few reported cases in the literature [1]. We report a 78-year-old male with end-stage renal disease (ESRD) on hemodialysis who developed L4-L5 discitis caused by *K. aerogenes*. Initial blood cultures and disc aspiration were negative, requiring repeat aspiration for diagnosis. The patient received intravenous cefepime during a two-week inpatient course, followed by a switch to oral levofloxacin to complete a total of eight weeks of antimicrobial therapy. This case highlights the importance of considering Gram-negative organisms in vertebral osteomyelitis in hemodialysis patients and the value of repeat sampling when initial cultures are nondiagnostic.

Vertebral osteomyelitis is a serious infectious condition whose incidence has increased, particularly among elderly patients with significant comorbidities [2]. Patients with ESRD receiving hemodialysis are at high risk because of immune dysfunction, recurrent bacteremia associated with vascular access, and frequent healthcare exposure [2-4]. Infectious spondylodiscitis has been reported to account for approximately 7% of hospitalized cases in the hemodialysis population, with vascular access identified as the presumed source of infection in up to 31% of patients [4].

*S. aureus* accounts for approximately half of vertebral osteomyelitis cases, but Gram-negative organisms are increasingly recognized, particularly in healthcare-associated infections [2,5]. *K. aerogenes *(formerly *Enterobacter aerogenes*) is an opportunistic Gram-negative bacterium that rarely causes vertebral osteomyelitis [1]. In healthcare-associated vertebral osteomyelitis, Enterobacteriaceae have been isolated in 15% of cases, with higher rates in patients with chronic renal failure [5].

Diagnosis can be challenging due to nonspecific symptoms and negative initial cultures, which occur in approximately 23% of cases [6]. The Infectious Diseases Society of America (IDSA) recommends withholding empiric antibiotics until microbiologic diagnosis is confirmed, except in septic or neurologically compromised patients [6]. Image-guided aspiration or biopsy is essential when blood cultures are negative, and repeat sampling may be necessary when initial attempts are non-diagnostic [6,7].

## Case presentation

We present a 78-year-old male with a past medical history of ESRD on hemodialysis through a left arm fistula, presenting to the Emergency Department with a three-day history of generalized weakness, nausea, and abdominal pain. Prior to the presentation, the patient had been feeling unwell for several weeks. He denied fever, vomiting, or dysuria. He had a left brachiobasilic fistula first placed in October 2025. The fistula was functional but was too medial and deep to use; therefore, he underwent revision with transposition and re-anastomosis in January 2026. Upon further probing, the patient mentioned that he had been experiencing new-onset back pain for the past two months, coinciding with the vascular manipulation.

On arrival, vital signs revealed a temperature of 101.5°F, heart rate of 110 bpm, blood pressure of 199/86 mmHg, and oxygen saturation of 92% on room air. Physical examination demonstrated lumbar spine tenderness and decreased bilateral basilar air entry on chest exam. Laboratory studies were notable for elevated white blood cell count, decreased hemoglobin, elevated erythrocyte sedimentation rate, and elevated C-reactive protein (Table 1). Urinalysis was unremarkable. The patient was started on intravenous vancomycin and intravenous cefepime by the emergency department after obtaining two sets of blood cultures.

**Table 1:** Laboratory parameters on admission. Summary of pertinent laboratory values obtained upon presentation to the Emergency Department.

Parameter	Value	Reference Range	Interpretation
White blood cell count	20.6 × 10³/μL	4.5-11.0 × 10³/μL	Elevated
Hemoglobin	9.9 g/dL	13.5-17.5 g/dL	Decreased
Erythrocyte sedimentation rate	67 mm/hr	0-20 mm/hr	Elevated
C-reactive protein	16.5 mg/L	0-5.0 mg/L	Elevated

Chest X-ray showed bilateral pleural effusions, more prominent on the left lung (Figure 1). The bilateral pleural effusions were attributed to fluid overload in the setting of ESRD and were not considered infectious in origin; sputum cultures were not obtained as the patient had no respiratory symptoms, such as cough or sputum production, and the clinical presentation was not suggestive of a primary pulmonary infectious process. CT of the abdomen and pelvis revealed L4-L5 discitis with severe canal stenosis and large bilateral pleural effusions. MRI confirmed L4-L5 discitis without evidence of epidural extension or paravertebral abscess (Figure 2). Blood cultures remained negative after five days.

**Figure 1:**
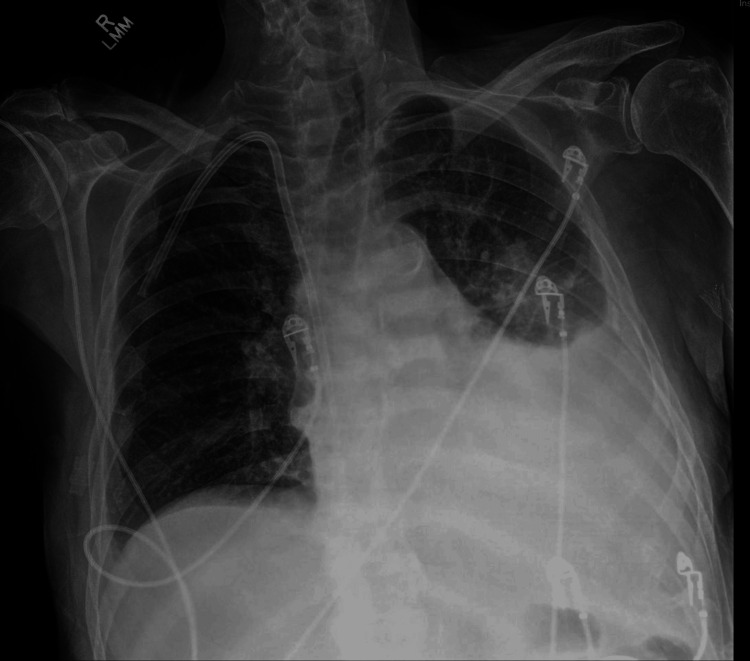
Posteroanterior chest radiograph demonstrating bilateral pleural effusions, moderate-to-severe on the left and mild on the right.

**Figure 2:**
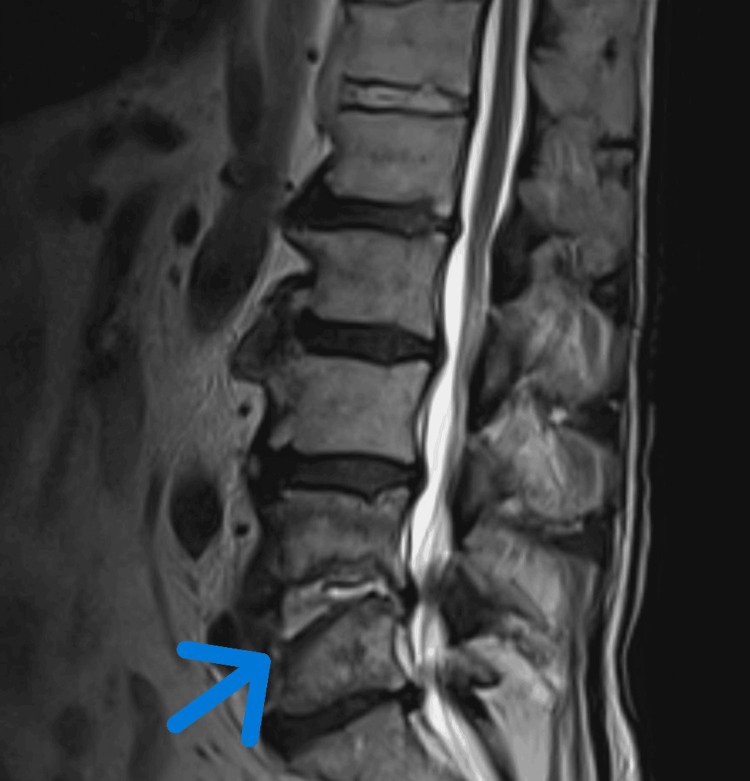
MRI of the lumbar spine demonstrating L4-L5 discitis. The blue arrow indicates the area of disc space infection at the L4-L5 level, characterized by abnormal signal intensity consistent with discitis. No evidence of epidural extension or paravertebral abscess was identified.

Following the consultation of the Infectious Diseases team, empiric antibiotics were held pending microbiologic diagnosis. Initial CT-guided disc space aspiration performed by the Interventional Radiology team after 48 hours of holding antibiotics yielded no culture growth. Given high clinical suspicion, a repeat aspiration after an additional 48 hours was performed, again with no culture growth. The Orthopedics team was consulted for a bone biopsy, which grew *K. aerogenes*. The biopsy specimen was submitted for aerobic and anaerobic cultures. Organism identification and antimicrobial susceptibility testing were performed using the VITEK 2 automated system.

Antimicrobial susceptibility testing results are summarized in Table 2. Notably, cefoxitin resistance (MIC ≥ 64 μg/mL) was identified by the VITEK 2 system and interpreted according to the Clinical and Laboratory Standards Institute (CLSI) guidelines as consistent with AmpC β-lactamase production, a chromosomally mediated resistance mechanism characteristic of *K. aerogenes *[8]. Intravenous cefepime, which is stable against AmpC β-lactamases [9], was initiated based on susceptibility results. The patient remained hemodynamically stable during the hospital stay. He was eventually discharged with outpatient oral levofloxacin therapy at a loading dose of 750 mg, followed by 500 mg every 48 hours. The patient reported significant clinical improvement upon further outpatient infectious disease follow-up.

**Table 2:** Antimicrobial susceptibility testing - Klebsiella aerogenes. Summary of minimum inhibitory concentrations (MICs) and susceptibility interpretations for the *Klebsiella aerogenes *isolate obtained from CT-guided bone biopsy of the L4-L5 disc space.

Antibiotic	MIC (μg/mL)	Interpretation
Cefepime	≤0.12	Susceptible
Cefoxitin	≥64	Resistant
Ceftriaxone	≤0.25	Susceptible
Ciprofloxacin	≤0.06	Susceptible
Gentamicin	≤1	Susceptible
Levofloxacin	≤0.12	Susceptible
Trimethoprim-sulfamethoxazole	≤20	Susceptible

## Discussion

This case illustrates *K. aerogenes* as an uncommon cause of discitis in hemodialysis patients and highlights several important diagnostic and management considerations. Patients receiving HD are at increased risk of vertebral osteomyelitis because of recurrent vascular access manipulation, frequent healthcare exposure, and immune dysfunction [3,4]. Native vertebral osteomyelitis typically results from hematogenous seeding, and patients on hemodialysis are particularly vulnerable because of recurrent bloodstream exposure from vascular access, repeated invasive procedures, and frequent healthcare contact [10]. A genitourinary source could not be excluded despite an unremarkable urinalysis and normal CT abdomen/pelvis findings, as *Klebsiella *species are well-known colonizers of the urinary tract and a recognized source of Gram-negative bacteremia.

In hemodialysis cohorts, bacteremia and vascular access manipulation have been identified as major risk factors, and vascular-access-associated infection has been implicated in approximately one-third of cases. In parallel, ESRD is associated with impaired innate and adaptive immune responses, including neutrophil dysfunction, T-cell abnormalities, B-cell lymphopenia, and chronic systemic inflammation, all of which may impair clearance of bacteremia and facilitate metastatic seeding of the spine. Diagnosis may be further delayed because spondylodiscitis in hemodialysis patients often presents with prolonged back pain with or without fever.

The diagnostic challenge in this case was compounded by negative blood cultures and negative initial disc aspiration cultures. Approximately 23% of vertebral osteomyelitis cases have negative blood cultures [6]. The Infectious Diseases Society of America (IDSA) guidelines recommend image-guided aspiration when blood cultures are negative or do not grow *S. aureus, Staphylococcus lugdunensis, *or *Brucella* [6,7]. The sensitivity of disc space aspiration varies, with positive cultures in approximately 56% of cases. When initial aspiration is non-diagnostic, repeat image-guided biopsy or percutaneous endoscopic discectomy should be considered [6]. Despite two negative CT-guided disc aspirations, the decision to pursue open bone biopsy was guided by the persistence of clinical and radiographic findings highly suggestive of infection. The patient continued to exhibit elevated inflammatory markers (ESR: 67 mm/hr, CRP: 16.5 mg/L), MRI findings demonstrating L4-L5 disc space enhancement consistent with discitis, and ongoing clinical symptoms.

The decision to withhold antibiotics pending microbiologic diagnosis, as recommended by the IDSA guidelines, likely contributed to successful organism isolation [6]. This approach is appropriate in hemodynamically stable patients without neurologic compromise and allows for targeted antimicrobial therapy based on susceptibility testing.

The IDSA recommends six weeks of parenteral or highly bioavailable oral antimicrobial therapy for most patients with bacterial vertebral osteomyelitis [6]. This recommendation is based on a randomized trial demonstrating non-inferiority of six weeks compared to 12 weeks, with cure rates of 90.9% in both groups [11]. Our patient received eight weeks of therapy, which falls within an acceptable range and may reflect consideration of his ESRD status and multiple comorbidities.

Outcomes in hemodialysis patients with vertebral osteomyelitis require careful monitoring. In a cohort of 102 hemodialysis patients with infectious spondylodiscitis, in-hospital survival was 82.4%, one-year survival was 78.4%, and a one-year recurrence rate was 20.2% [4]. Congestive heart failure was associated with increased mortality, while most other variables did not predict outcomes [4]. Our patient's favorable response with resolution of fever and improvement in inflammatory markers is encouraging, though close monitoring with serial imaging and inflammatory markers remains essential to detect treatment failure or recurrence [6].

Although *K. aerogenes* is a rare cause of vertebral osteomyelitis, Gram-negative organisms as a group are increasingly recognized in this setting, particularly in healthcare-associated infections and in patients with chronic renal failure [1,12-15]. Table 3 summarizes published case reports and series of Gram-negative spondylodiscitis/vertebral osteomyelitis, including cases caused by *Klebsiella *species and other Gram-negative bacilli. Notably, the only other reported case of *K. aerogenes* spinal infection was diagnosed using metagenome next-generation sequencing (mNGS), whereas the present case required repeat bone biopsy - highlighting the variability in diagnostic approaches for this rare pathogen.

**Table 3:** Summary of the selected published cases of Gram-negative vertebral osteomyelitis: causative organisms, clinical presentations, treatments, and outcomes. ESR: end-stage renal disease; GNB: Gram-negative bacilli; mNGS: metagenome next-generation sequencing

Author (Year)	Organism	Patient	Presentation	Diagnosis	Treatment	Outcome
Gu et al. (2022) [1]	K. aerogenes	China, elderly	Low back pain, fever	mNGS of disc tissue	IV antibiotics	Improved
Inagaki et al. (2019) [13]	K. pneumoniae	Japan, adult male	Back pain, bacteremia	Blood cultures, MRI	IV antibiotics	Cured
Park et al. (2014) [12]	GNB (various)	Korea, 57 patients	Back pain ± fever	Blood/tissue cultures	IV antibiotics 6-8 wks	86% cure
Graham et al. (2013) [15]	GNB (various)	UK, 15 patients	Back pain, fever	Blood/tissue cultures	IV antibiotics 6-12 wks	87% cure
Pigrau et al. (2015) [5]	Enterobacteriaceae	Spain, HA-VO series	Back pain ± fever	Blood/tissue cultures	Targeted IV therapy	Variable
Cebrián Parra et al. (2012) [14]	Mixed (incl. GNB)	Spain, 108 patients	Back pain, elevated ESR/CRP	Biopsy cultures	IV then oral antibiotics	89% cure
Present case (2026)	K. aerogenes	USA, 78M, HD	Back pain, fever, weakness	Bone biopsy	IV then oral antibiotics	Improved

## Conclusions

This case demonstrates *K. aerogenes* as a rare but important cause of discitis in hemodialysis patients. Gram-negative organisms should be considered in the differential diagnosis of vertebral osteomyelitis in this population, particularly following vascular access procedures. Negative initial blood cultures and image-guided aspirations should not preclude further diagnostic evaluation when clinical suspicion remains high. Repeat sampling may be necessary to establish microbiologic diagnosis and guide targeted antimicrobial therapy. Management requires a multidisciplinary approach with antimicrobial selection based on susceptibility testing and treatment duration of six to eight weeks for uncomplicated cases. Early recognition and appropriate management are essential to prevent disease progression, treatment failure, and recurrence in this high-risk population.
